# Targeting ERβ1-Positive Triple-Negative Breast Cancer: Molecular Effects of Calcitriol and 17β-Estradiol

**DOI:** 10.7759/cureus.82934

**Published:** 2025-04-24

**Authors:** Shankaramurthy K N, Praveenkumar Shetty, Basavaraj Devaranagadi, Indira A Hundekari

**Affiliations:** 1 Biochemistry, Bijapur Lingayat District Educational (BLDE) Shri B.M. Patil Medical College Hospital and Research Centre, Vijayapura, IND; 2 Biochemistry, K S Hegde Medical Academy, NITTE (Deemed to be University), Mangaluru, IND

**Keywords:** 17β-estradiol, breast cancer, calcitriol, caspase 3, cytotoxicity, egfr, erβ1, hormone-based therapy, tnbc, vegf

## Abstract

Background

Breast cancer is the most common malignancy in women. Triple-negative breast cancer (TNBC) is characterized by the absence of estrogen receptor, progesterone receptor, and human epidermal growth factor receptor 2, contributing to its aggressive nature, limited treatment options, and poor prognosis. Emerging evidence highlights estrogen receptor beta 1 (ERβ1) as a potential tumor suppressor in TNBC, influencing key oncogenic pathways such as cell proliferation, survival, angiogenesis, and apoptosis. In this regard, calcitriol (active vitamin D) and 17β-estradiol have been identified as key regulators of tumor behavior. Calcitriol shows strong anti-proliferative and pro-apoptotic effects, while the ability of 17β-estradiol to modulate tumor progression through ERβ1 signaling is context-dependent. This study aims to investigate the individual and combined effects of calcitriol and 17β-estradiol in ERβ1-expressing MDA-MB-468 TNBC cells, with a focus on their role in regulation of tumor progression, angiogenesis, and apoptosis. The findings provide novel insights into the potential therapeutic utility of targeting ERβ1 in TNBC.

Methodology

The MDA-MB-468 TNBC cells were treated with calcitriol (1-5 µM) and/or 17β-estradiol (100-500 nM). The effect on cell viability was assayed with the 3-(4,5-dimethylthiazol-2-yl)-2,5-diphenyltetrazolium bromide (MTT) method. At the same time, immunoblot analysis investigated the time-dependent manner of ERβ1, epidermal growth factor receptor (EGFR), vascular epithelial growth factor (VEGF), and caspase-3.

Results

Both calcitriol and 17β-estradiol substantially decreased TNBC cell viability, with the highest level of cytotoxicity observed at 24 and 32 hours, respectively. The combination increased the amount of cell death. Immunoblots revealed lasting downregulation of ERβ1, EGFR, VEGF, and caspase-3 after calcitriol treatment. In comparison, 17β-estradiol demonstrated biphasic regulatory behavior for ERβ1, where ERβ1 was first downregulated, then partially recovered. The combination therapies produced more significant ERβ1 downregulation and heightened suppression of EGFR and VEGF, further enhancing their effects on TNBC progression.

Conclusions

This study aims to investigate the individual and combined effects of calcitriol and 17β-estradiol in ERβ1-expressing MDA-MB-468 TNBC cells, with a focus on their role in the regulation of tumor progression, angiogenesis, and apoptosis.

## Introduction

Breast cancer (BC) is the most frequently diagnosed cancer in women globally [1]. Among its subtypes, triple-negative breast cancer (TNBC) is the most aggressive, comprising 15-20% of BC cases [2]. TNBC is characterized by a lack of estrogen receptor (ER), progesterone receptor (PR), and human epidermal growth factor receptor 2 (HER2) expression, consequently making TNBC the most challenging subtype to treat due to a lack of targeted treatment options [3]. This is more common in younger women, African Americans, and those with *BRCA1* mutations [4]. Currently, chemotherapy is the standard treatment, but the high rate of recurrences, the onset of chemoresistance, and poor survival indicate an urgent need for new therapeutic options [5,6]. Recently, estrogen receptor beta 1 (ERβ1) has emerged as a potential tumor suppressor in TNBC and regulates oncogenic pathways essential for proliferation, survival, angiogenesis, and apoptosis [7,8]. While ERα drives tumorigenesis in hormone receptor-positive BC, ERβ1 is thought to function in an anti-tumorigenic manner, and the expression of ERβ1 in a group of TNBC cases provides a novel therapeutic avenue [9]. However, despite its potential, the therapeutic implications of targeting ERβ1 in TNBC remain underexplored [10]. Calcitriol and 17β-estradiol have been investigated for their roles in modulating BC cell behavior. Calcitriol exerts anti-proliferative and pro-differentiation effects, while 17β-estradiol can have context-dependent effects on tumor progression, depending on receptor expression and pathway activation [11,12]. Although both compounds have shown promising effects, their interaction with ERβ1’s exact molecular mechanisms remains unclear, particularly in TNBC.

This study aims to investigate the individual and combined effects of calcitriol and 17β-estradiol on TNBC using the ERβ1-positive MDA-MB-468 cell line. The focus is on their time-dependent effects on key regulatory proteins involved in tumor progression, including epidermal growth factor receptor (EGFR) for proliferation, vascular endothelial growth factor (VEGF) for angiogenesis, and caspase-3 for apoptosis. This study contributes to the development of hormone-based therapeutic strategies for TNBC, particularly in ERβ1-positive tumors, by highlighting the regulatory effects of calcitriol and 17β-estradiol on key oncogenic pathways.

## Materials and methods

Cell line and reagents

The MDA-MB-468 TNBC cell line was obtained from the National Centre for Cell Science (NCCS), Pune, India, and maintained in Leibovitz’s L-15 medium supplemented with 10% fetal bovine serum and 1% antibiotic-antimycotic solution. 3-(4,5-dimethylthiazol-2-yl)-2,5-diphenyltetrazolium bromide (MTT) and dimethyl sulfoxide (DMSO) were also used. All cell culture reagents were procured from HiMedia Laboratories Pvt. Ltd., Mumbai, India. Cells were cultured at 37°C in a humidified incubator without CO₂. Calcitriol (Cat. No. S1466) was purchased from Selleckchem (Houston, TX, USA), and 17β-estradiol was obtained from Sigma-Aldrich (St. Louis, MO, USA).

Evaluation of cell viability

MTT assay was employed to determine cell viability [13] after treatment with calcitriol and 17β-estradiol. MDA-MB-468 cells were seeded at 2 × 10⁴ cells per well in 96-well plates and left overnight for attachment. The cells were treated with calcitriol at 1, 2, 3, 4, and 5 µM, or with 17β-estradiol at 100, 200, 300, 400, and 500 nM. A combination treatment consisting of 5 µM calcitriol and 500 nM 17β-estradiol was also included. Control cells were not treated. After 8, 16, 24, and 32 hours of treatment, each well received 10% MTT solution and was incubated for four hours at 37°C in a 5% CO₂ humidified atmosphere. The resulting formazan diagnostic crystals were dissolved in DMSO, and absorbance was quantified at 570 nm using a Cytation microplate reader (BioTek Instruments Inc., Winooski, VT, USA). Optical density (OD) values were recorded to quantify relative cell viability. The percentage of cell viability was calculated using the following formula: % cell viability = (Absorbance of treated cells/Absorbance of control cells) × 100 [13].

Immunoblot analysis

Western blot analysis was performed to assess the expression of ERβ1, EGFR, VEGF, and caspase-3 following treatment. After treatment, cell protein homogenates (20 μg per well) were subjected to separation by electrophoresis using 10% sodium dodecyl sulfate-polyacrylamide gel electrophoresis and subsequently transferred onto nitrocellulose membranes (HiMedia Laboratories Pvt. Ltd., Mumbai, India). To minimize non-specific binding, the membranes were blocked with 2% bovine serum albumin in tris-buffered saline and incubated overnight at 4°C with monoclonal antibodies specific to ERβ1 (sc-390243), EGFR (sc-53274), VEGF (sc-7269), caspase-3 (sc-56046), and GAPDH (sc-137179), all obtained from Santa Cruz Biotechnology, CA, USA. Afterwards, the membrane was incubated at room temperature for two hours in the presence of horseradish peroxidase-conjugated secondary antibodies (sc-2031, Santa Cruz Biotechnology).

Protein detection was performed using the enhanced chemiluminescence (ECL) substrate (Bio-Rad, UK), and protein bands were documented using the SYNGENE G: G-Box Chemi-XR5 imaging system (GENESYS V1.4.1.0). The intensities of the protein bands were quantified using ImageJ software, with expression levels normalized to GAPDH to ensure equal protein loading. Background subtraction and normalization were performed to improve quantification accuracy, ensuring reliable comparisons between treatment groups.

Statistical analysis

Cell viability assays were performed using at least three independent replicates (n = 3). Data were analyzed as mean ± standard error of the mean (SEM). Statistical analysis was performed with one-way analysis of variance (ANOVA), using Tukey’s post-hoc test to assess multiple treatment groups. P-values ≤0.05 were considered statistically significant. Data visualization and graphs were made using GraphPad Prism version 8.

## Results

**Figure 1:**
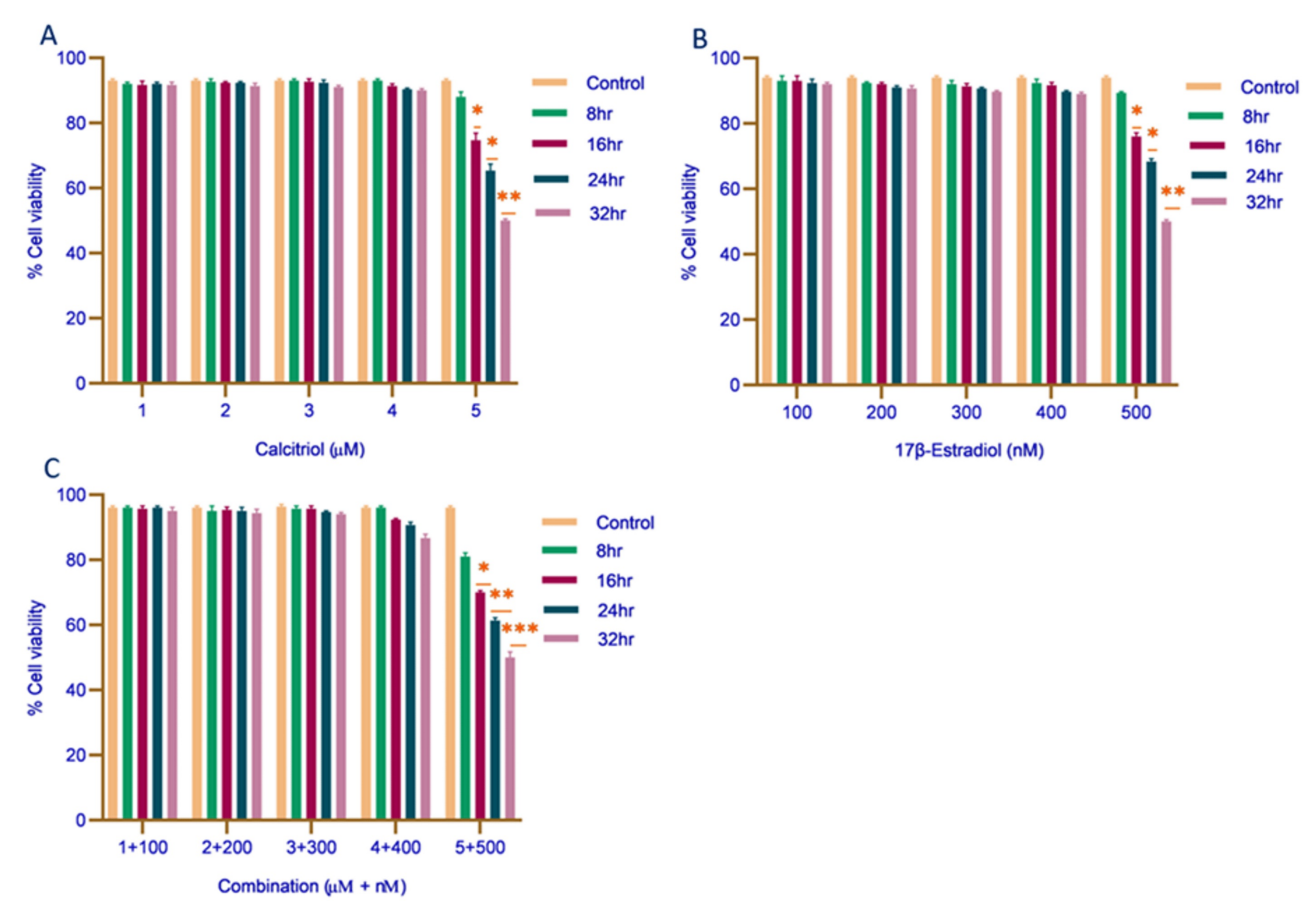
Effect of calcitriol, 17β-estradiol, and its combination on MDA-MB-468 cell viability. Cell viability was assessed using the MTT assay following treatment with (A) Calcitriol (1, 2, 3, 4, and 5 μM), (B) 17β-estradiol (100, 200, 300, 400, and 500 nM), and (C) combination treatment (calcitriol, 5 μM + 17β-estradiol, 500 nM) at the indicated concentrations for 8, 16, 24, and 32 hours. Data are expressed as mean ± SEM from three independent experiments. Statistically significant differences in cell viability compared to the untreated control at each time point are indicated (*p < 0.01, **p < 0.001, *p < 0.0001). Statistical analysis was performed using one-way ANOVA followed by Tukey’s post hoc test. SEM: standard error mean; μM: micromolar; nM: nanomolar; hr: hours

The cytotoxic effects of calcitriol, 17β-estradiol, and their combination on MDA-MB-468 cell viability were assessed using the MTT assay. Calcitriol treatment (1, 2, 3, 4, and 5 µM) resulted in a significant, time-dependent reduction in cell viability, with the most pronounced decreases observed at 16 hours (74%), 24 hours (65%), and 32 hours (50%), as shown in Figure 1A. Similarly, 17β-estradiol treatment (100, 200, 300, 400, and 500 nM) induced a substantial decline in viability, with the greatest reductions observed at 16 hours (76%), 24 hours (68%), and 32 hours (50%), as illustrated in Figure 1B. Notably, the combination treatment exhibited an additive cytotoxic effect, further reducing cell viability at 16 hours (70%), 24 hours (61%), and 32 hours (50%), as represented in Figure 1C, indicating an enhanced suppression compared to individual treatments. The most substantial reductions were observed between 24 and 32 hours, suggesting a delayed but sustained inhibitory effect on TNBC cell survival. These findings demonstrated that calcitriol and 17β-estradiol exert cytotoxic effects in a time-dependent manner, with their combination therapy potentially enhancing TNBC cell suppression.

Immunoblot analysis

Effects of Calcitriol Treatment in MDA-MB-468 Cells

**Figure 2:**
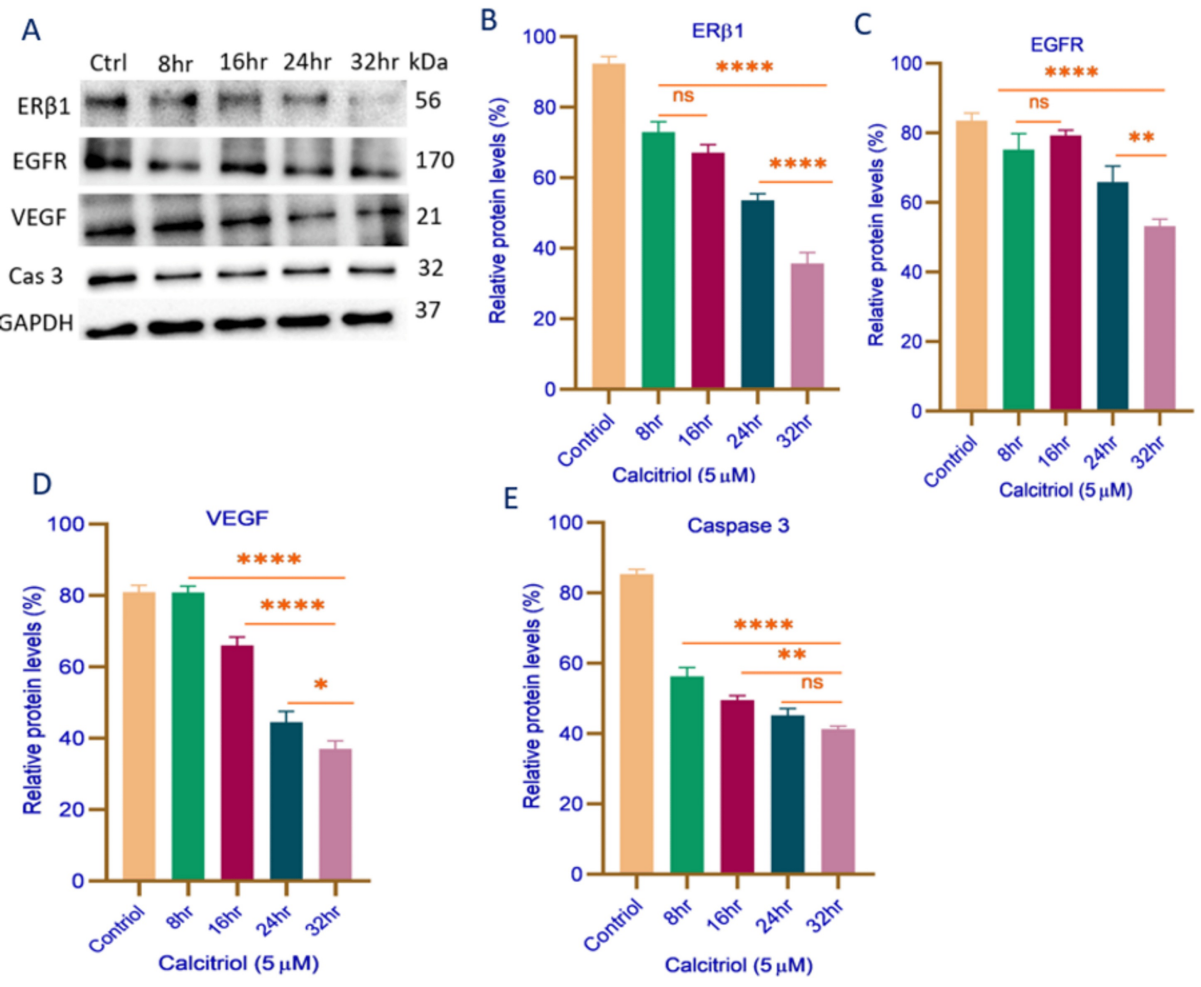
Representative Western blot analysis (A) and quantification of protein expression levels of ERβ1 (B), EGFR (C), VEGF (D), and caspase-3 (E) in MDA-MB-468 cells treated with 5 µM calcitriol at different time intervals (8, 16, 24, and 32 hours). Protein expression levels were normalized to GAPDH and are presented as relative percentages. Data are expressed as mean ± SD. Statistical significance is indicated as *p < 0.01, **p < 0.005, ***p < 0.001, ****p < 0.0001. Statistical analysis was performed using one-way ANOVA followed by Tukey’s post hoc test. ERβ1: estrogen receptor beta 1; EGFR: epidermal growth factor receptor; VEGF: vascular endothelial growth factor; Cas 3: caspase 3; GAPDH: glyceraldehyde phosphate dehydrogenase; Ctrl: control; hr: hours; kDa: kilodalton; μM: micromolar; ns: not significant

ERβ1 expression in MDA-MB-468 cells exhibited a time-dependent suppression following treatment with 5 μM calcitriol. As a tumor suppressor in TNBC, ERβ1 plays a pivotal role in regulating cell proliferation and survival pathways. No statistically significant reduction was observed between 8 and 16 hours (p = ns); however, a progressive decline was detected from 8 to 32 hours (p < 0.0001), indicating sustained suppression over time (Figure 2A). Furthermore, a significant additional reduction occurred between 24 and 32 hours (p < 0.0001), as illustrated in Figure 2B, suggesting a prolonged inhibitory effect at later time points. Similarly, EGFR expression, a key regulator of proliferative and survival signaling in TNBC, exhibited a time-dependent suppression. A significant reduction was observed from 8 to 32 hours (p < 0.0001), whereas no notable change was detected between 8 and 16 hours (p = ns). A further decrease in expression occurred between 24 and 32 hours (p = 0.005), as shown in Figure 2C, indicating a delayed but sustained inhibitory effect. Additionally, calcitriol treatment markedly reduced VEGF expression over time, with a statistically significant decrease from 8 to 32 hours (p < 0.0001). A further suppression was noted from 16 to 32 hours (p < 0.0001), though between 24 and 32 hours, VEGF expression exhibited a slight but statistically significant decline (p = 0.01), as depicted in Figure 2D. Lastly, caspase-3 expression progressively declined, with a significant reduction from 8 to 32 hours (p < 0.0001) and from 16 to 32 hours (p = 0.001). However, expression remained stable between 24 and 32 hours (p = ns), as presented in Figure 2E, indicating a potential regulatory threshold for apoptosis induction.

These findings collectively indicate that calcitriol exerts a sustained inhibitory effect on key regulatory proteins involved in TNBC proliferation, angiogenesis, and apoptosis, with the most substantial suppression occurring at extended time points.

Effects of 17β-Estradiol Treatment in MDA-MB-468 Cells

**Figure 3:**
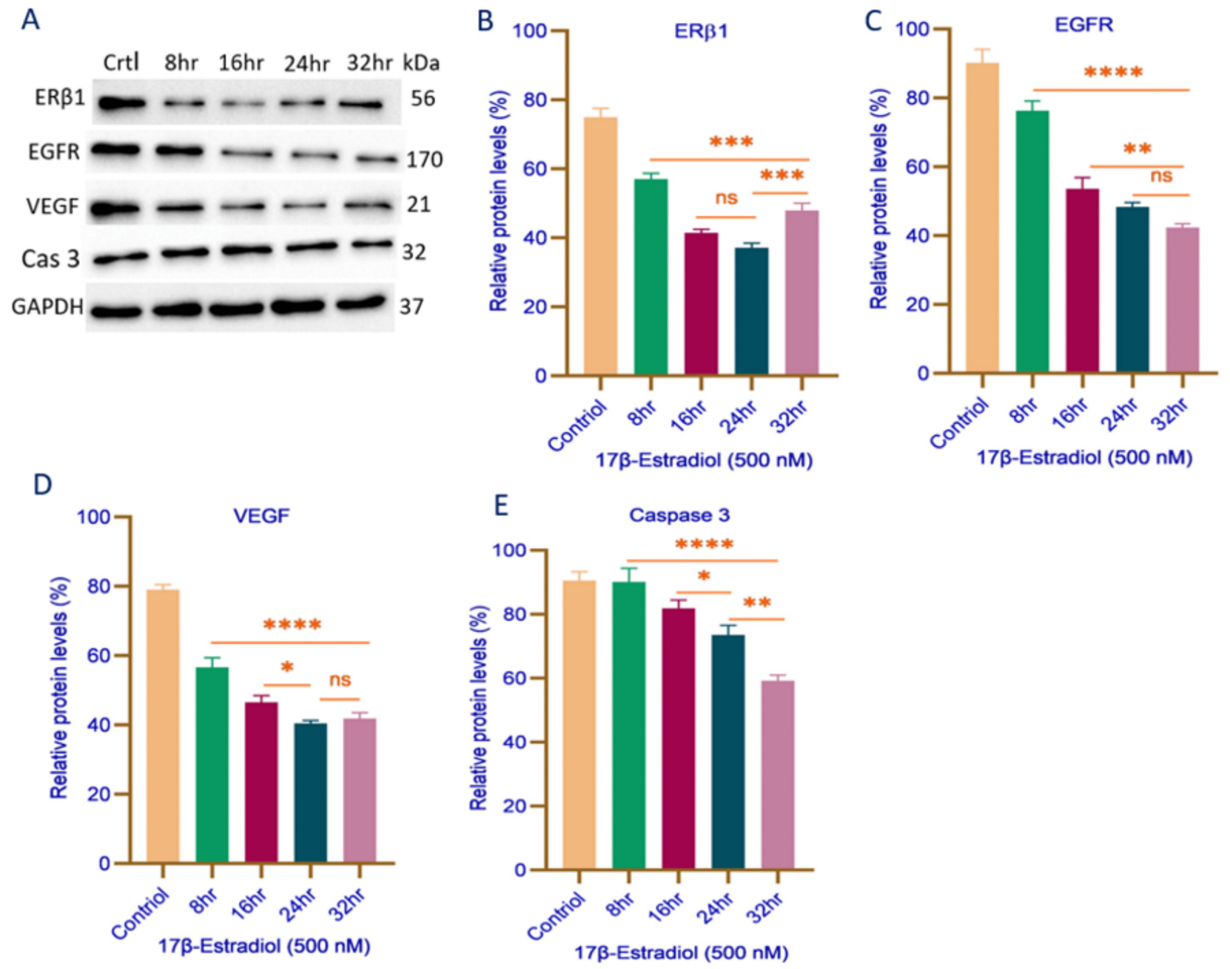
Representative Western blot analysis (A) and quantification of protein expression levels of ERβ1 (B), EGFR (C), VEGF (D), and caspase-3 (E) in MDA-MB-468 cells treated with 500 nM 17β-estradiol at different time intervals (8, 16, 24, and 32 hours). Protein expression levels were normalized to GAPDH and are presented as relative percentages. Data are expressed as mean ± SD. Statistical significance is indicated as *p < 0.01, **p < 0.003, ***p < 0.001, ****p < 0.0001. Statistical analysis was performed using one-way ANOVA followed by Tukey’s post hoc test. ERβ1: estrogen receptor beta 1; EGFR: epidermal growth factor receptor; VEGF: vascular endothelial growth factor; Cas 3: caspase 3; GAPDH: glyceraldehyde phosphate dehydrogenase; Ctrl: control; hr: hours; kDa: kilodalton; nM: nanomolar; ns: not significant

17β-estradiol (500 nM) treatment in MDA-MB-468 cells resulted in a time-dependent manner of key protein expressions. A statistically significant reduction was observed from 8 to 32 hours (p = 0.0009), indicating a progressive decline in expression over time (Figure 3A). However, between 16 and 24 hours, a slight reduction was noted, though it was not statistically significant (p = ns), suggesting a transient stabilization. Interestingly, from 24 to 32 hours, ERβ1 expression significantly increased (p = 0.0002), indicating a potential compensatory or adaptive response at later time points, as represented in Figure 3B. Similarly, EGFR expression exhibited a time-dependent suppression following treatment. A statistically significant decrease was observed from 8 to 32 hours (p < 0.0001), indicating a progressive decline in expression over time. Additionally, between 16 and 32 hours, EGFR expression was significantly reduced (p = 0.003), suggesting a sustained inhibitory effect. However, between 24 and 32 hours, a slight reduction was observed, though it was not statistically significant (p = ns), indicating a potential stabilization of the inhibitory response at later time points, as illustrated in Figure 3C.

VEGF expression exhibited a time-dependent suppression following treatment. A statistically significant reduction was observed from 8 to 32 hours (p < 0.0001), indicating a progressive decline in expression over time. Additionally, between 16 and 24 hours, VEGF expression was significantly reduced (p < 0.01), suggesting a continued inhibitory effect at intermediate time points. However, between 24 and 32 hours, a slight reduction was observed, though it was not statistically significant (p = ns), indicating a potential stabilization of VEGF suppression at later stages, as shown in Figure 3D. Caspase-3 expression exhibited a time-dependent suppression following treatment. A statistically significant reduction was observed from 8 to 32 hours (p = 0.0001), indicating a progressive decline in apoptotic marker expression over time. Additionally, between 16 and 32 hours, a moderate reduction in expression was noted (p = 0.003), suggesting a sustained but gradual inhibitory effect. Furthermore, between 24 and 32 hours, caspase-3 expression was significantly reduced (p = 0.001), highlighting a pronounced suppression at later time points, as depicted in Figure 3E. These findings suggest that 17β-estradiol regulates ERβ1, EGFR, VEGF, and caspase-3 expression in a time-dependent manner, influencing proliferation, angiogenesis, and apoptosis in MDA-MB-468 cells.

Combination Effects of Calcitriol and 17β-Estradiol in MDA-MB-468 Cells

**Figure 4:**
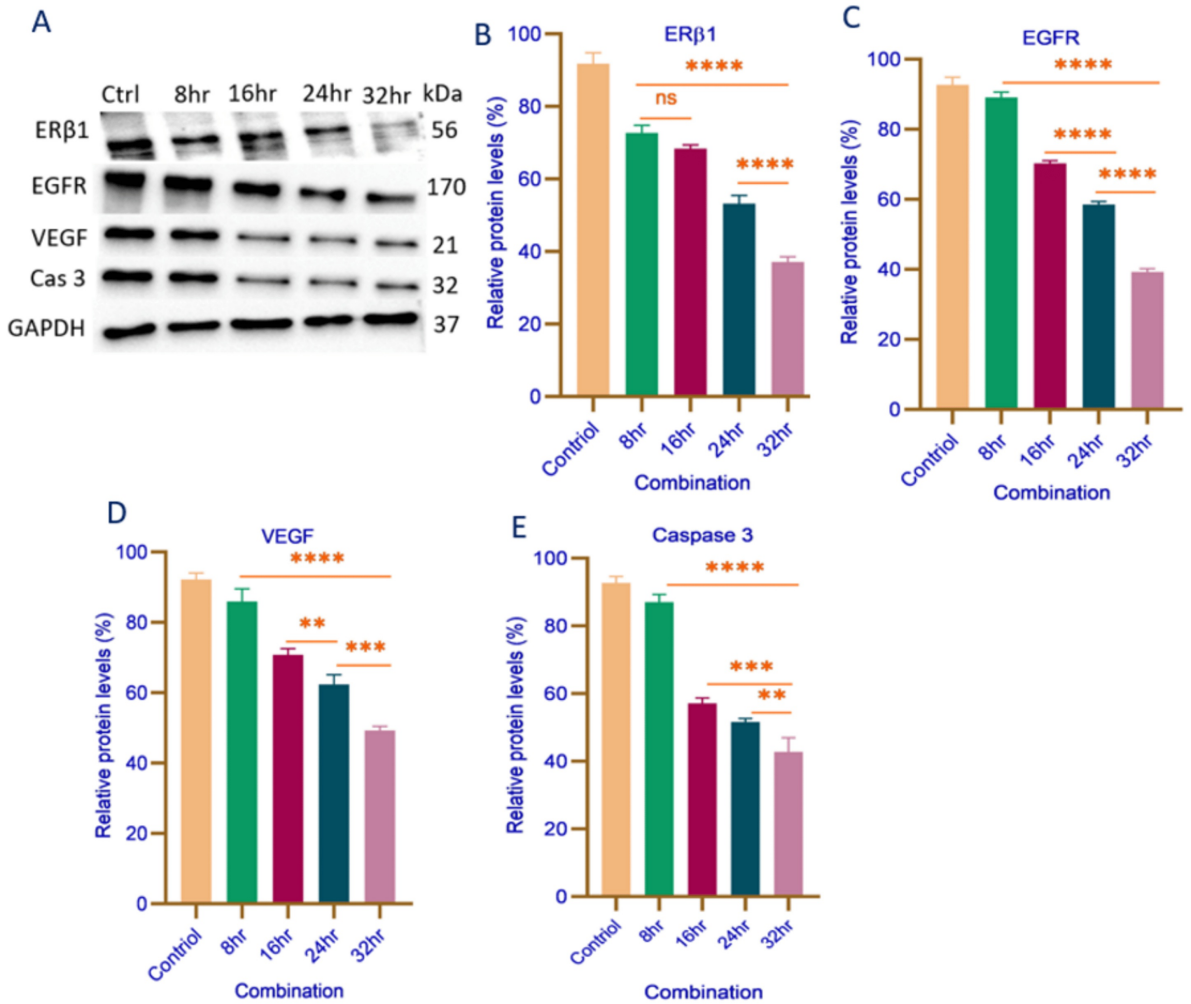
Representative Western blot analysis (A) and quantification of protein expression levels of ERβ1 (B), EGFR (C), VEGF (D), and caspase-3 (E) in MDA-MB-468 cells treated with the combination of calcitriol (5 µM) and 17β-estradiol (500 nM) at different time intervals (8, 16, 24, and 32 hours). Protein expression levels were normalized to GAPDH and are presented as relative percentages. Data are expressed as mean ± SD. Statistical significance is indicated as **p < 0.008, ***p < 0.004, ****p < 0.0001. Statistical analysis was performed using one-way ANOVA followed by Tukey’s post hoc test. ERβ1: estrogen receptor beta 1; EGFR: epidermal growth factor receptor; VEGF: vascular endothelial growth factor; Cas 3: caspase 3; GAPDH: glyceraldehyde phosphate dehydrogenase; Ctrl: control; hr: hours; kDa: kilodalton; ns: not significant

The combination treatment of calcitriol (5 µM) and 17β-estradiol (500 nM) in MDA-MB-468 cells resulted in an enhanced downregulation of key regulatory proteins. ERβ1 expression exhibited a statistically significant reduction from 8 to 32 hours (p = 0.0001). However, between 8 and 16 hours, no significant reduction was observed, suggesting an initial resistance (Figure 4A). Furthermore, from 24 to 32 hours, ERβ1 expression was significantly reduced (p = 0.0001), as shown in Figure 4B, indicating a progressive and sustained downregulation at later time points. Similarly, EGFR expression demonstrated a significant decline across all evaluated intervals: 8 to 32 hours, 16 to 24 hours, and 24 to 32 hours (p = 0.0001 for each), as shown in Figure 4C. These findings suggest that the combination treatment exerts a sustained inhibitory effect on ERβ1 and EGFR expression over time. VEGF expression also exhibited a significant decline from 8 to 32 hours (p < 0.0001); between 16 and 24 hours, the reduction was significant (p = 0.009), and further substantial decreases were observed between 24 and 32 hours (p = 0.0004), as illustrated in Figure 4D. Caspase-3 expression was significantly reduced from 8 to 32 hours (p < 0.0001), with additional significant decreases between 16 and 32 hours (p = 0.0002), and 24 and 32 hours (p = 0.008), as shown in Figure 4E. These findings indicate that a combination of calcitriol and 17β-estradiol provides more pronounced inhibition of ERβ1, EGFR, VEGF, and caspase-3 expressions in MDA-MB-468 cells over time.

## Discussion

Calcitriol and 17β-estradiol have received considerable attention regarding their roles in the regulation of cellular processes associated with cancer. In particular, both compounds interact with critical pathways regulating proliferation, apoptosis, and cancer progression. The current study indicates that treatment with calcitriol reduces MDA-MB-468 cell viability in a time-dependent manner, with the most significant effect at 16, 24, and 32 hours. This finding is consistent with past studies that have shown that vitamin D induces cytotoxicity in BC cells. For example, Bajbouj et al. observed a marked decrease in MDA-MB-231 cell viability after treatment with high doses of vitamin D, leading to a substantial reduction in cell survival [14]. Similarly, 17β-estradiol led to a significant decline in MDA-MB-468 cell viability, with major reductions at 16, 24, and 32 hours, consistent with prior reports on estrogen signaling in TNBC. Wang et al. demonstrated that MDA-MB-468 cells stably transfected with ERβ exhibited over 60% inhibition of proliferation upon treatment with 10 nM 17β-estradiol, supporting the role of ERβ activation in regulating TNBC cell viability [15]. In our study, the combination of calcitriol and 17β-estradiol exerted an additive cytotoxic effect, further reducing cell viability across all tested time points.

The modulation of ERβ1 by calcitriol in ERβ1-positive TNBC cells reflects a time-dependent regulatory pattern. In the present study, calcitriol treatment led to a gradual decline in ERβ1 expression over time, with significant suppression observed between 24 and 32 hours. This progressive downregulation is consistent with earlier reports indicating that nuclear hormone receptors, including ERβ1, are subject to ligand-induced regulation. Swami et al. have shown that activation of the vitamin D receptor by calcitriol can suppress ER signaling through transcriptional repression or post-translational degradation, indicating that ERβ1 may be regulated by similar mechanisms in TNBC [16]. Further analysis of ERβ1 expression following 17β-estradiol treatment revealed a biphasic response. ERβ1 expression remained unchanged at 8 hours, followed by a slight reduction at 16 hours and significant downregulation at 24 and 32 hours. This biphasic modulation suggests an initial transient activation followed by a feedback-driven downregulation. BC models have reported such patterns [17,18]. Previous studies indicate that ER expression is subject to dynamic control mechanisms, where ligand binding initially stabilizes receptor levels, followed by subsequent degradation or transcriptional repression [19]. The delayed ERβ1 downregulation observed in our study could be attributed to ligand-induced receptor turnover or negative feedback regulation mediated by downstream signaling cascades.

Furthermore, calcitriol induced time-dependent suppression of EGFR expression, with significant inhibition observed at the 24- and 32-hour time points. These results are consistent with previous studies showing that calcitriol and its analogs downregulate EGFR expression, thereby inhibiting proliferation and survival pathways in BC cells [20]. Interestingly, 17β-estradiol also caused time-dependent inhibition of EGFR, in line with reports suggesting that estrogen signaling may limit EGFR expression in TNBC. For example, Khode et al. observed a significant reduction in EGFR expression in MDA-MB-231 cells after treatment with 17β-estradiol, suggesting a link between estrogen signaling and growth factor receptor regulation [21].

Beyond its effects on proliferation, calcitriol significantly downregulated VEGF, a key regulator of angiogenesis. Marked reductions in VEGF levels were observed at 16 and 24 hours, with continued suppression at 32 hours, corroborating prior findings that calcitriol inhibits hypoxia-inducible factor-1/VEGF signaling in multiple tumor models [22]. Estrogen signaling has also been implicated in the regulation of angiogenesis. In the present study, VEGF expression exhibited a time-dependent decline following treatment with 17β-estradiol, with the most significant suppression observed between 16 and 24 hours. Although the reduction between 24 and 32 hours was not statistically significant, the overall trend indicates a sustained inhibitory effect, possibly reaching a plateau. These findings align with previous evidence showing that 17β-estradiol can suppress VEGF transcription in TNBC models via ERβ-mediated signaling, thereby limiting angiogenesis and tumor progression [23]. The observed early suppression followed by stabilization suggests that estrogenic modulation of VEGF is both time-sensitive and receptor-dependent.

In addition to angiogenic regulation, apoptotic signaling was also modulated. Calcitriol treatment led to a progressive decline in caspase-3 expression over time, with significant suppression observed from 8 to 32 hours and from 16 to 32 hours. These results suggest that calcitriol may initially activate apoptotic pathways, followed by a reduction in caspase-3 expression as downstream processes are engaged. The stabilization of caspase-3 levels between 24 and 32 hours may reflect feedback inhibition or completion of the apoptotic cascade. Prior studies have demonstrated that calcitriol induces apoptosis in TNBC cells via caspase-dependent mechanisms, including caspase-3 activation followed by proteolytic degradation [24]. Similarly, 17β-estradiol treatment resulted in a significant reduction in caspase-3 expression between 24 and 32 hours, indicating pronounced suppression at later time points. This delayed response may reflect ligand-receptor interactions that modulate caspase activity over time. Previous studies have reported that estradiol and its derivatives influence apoptosis through both caspase-dependent and -independent mechanisms in TNBC models [25]. While studies examining their combined effects in TNBC models remain limited, previous investigations have established their individual efficacy in inhibiting TNBC proliferation.

Overall, the findings provide novel insights into the combined effects of calcitriol and 17β-estradiol, highlighting their time-dependent regulation of key pathways associated with proliferation, angiogenesis, and apoptosis in ERβ1-positive TNBC.

Strengths and limitations

This study provides novel insights into the hormonal regulation of ERβ1-positive TNBC, demonstrating the cytotoxic and molecular effects of calcitriol and 17β-estradiol, both individually and in combination. A key strength of the study is the temporal analysis of multiple biomarkers, including ERβ1, EGFR, VEGF, and caspase-3, which allows for a comprehensive understanding of signaling pathways involved in proliferation, angiogenesis, and apoptosis. The evaluation of combination therapy may contribute to TNBC treatment by potentially inhibiting tumor progression.

However, the findings are currently limited to in vitro conditions, and further validation using animal models, such as TNBC mouse xenografts, is required to confirm the therapeutic potential of calcitriol and 17β-estradiol. While key regulatory proteins were analyzed, the precise molecular mechanisms underlying the observed effects remain to be fully elucidated. Future studies should investigate downstream signaling pathways related to hormone receptor activity, apoptotic regulation, and angiogenesis to enhance mechanistic understanding.

## Conclusions

This study demonstrates that calcitriol and 17β-estradiol regulate the expression of ERβ1, EGFR, VEGF, and caspase-3 in a time-dependent manner, suggesting their potential role in modulating key oncogenic pathways in ERβ1-positive TNBC. The observed regulatory effects on proliferation, angiogenesis, and apoptosis indicate that combination treatment with calcitriol and 17β-estradiol could be beneficial in targeting TNBC-associated signaling mechanisms. While the current findings are based on in vitro analysis, they provide a foundation for exploring the clinical applicability of hormone-based strategies in ERβ1-positive TNBC. Future studies should focus on delineating the molecular pathways involved in the observed combination effects, particularly those related to hormone receptor signaling, apoptotic regulation, and angiogenesis. Preclinical validation using TNBC mouse xenograft models will be essential to assess therapeutic relevance and confirm in vivo efficacy.
